# Harassment among Women at Workplace: A Cross-Sectional Study in Coastal South India

**DOI:** 10.4103/0970-0218.66888

**Published:** 2010-04

**Authors:** B Unnikrishnan, T Rekha, Ganesh Kumar, B Reshmi, P Mithra, B Sanjeev

**Affiliations:** Department of Community Medicine, Kasturba Medical College, Mangalore, (Manipal University), India; 1 Department of Community Medicine, JIPMER, Puducherry, India; 2 Department of Health Information Management, Manipal College of Allied Health Sciences, Manipal, India

## Introduction

Harassment is any improper and unwelcome conduct that might reasonably be expected or be perceived to cause offence or humiliation to another person. Harassment may take the form of words, gestures or actions which tend to annoy, alarm, abuse, demean, intimidate, belittle, humiliate or embarrass another or which create an intimidating, hostile or offensive work environment.(1) Women face discrimination from childhood, especially in communities where there is a preference for the male child.(2) Harassment in the work place is becoming increasingly important in all sectors of the economy, largely due to growing numbers of negative consequences. This has lead to the formulation of anti-harassment policies by several non-government organizations.(3)

This study was carried out to find out harassment among women at workplace, types and reasons for harassment generally faced by working women.

## Materials and Methods

A cross-sectional study was carried out within the Municipal Corporation limits of Mangalore, a coastal city in Karnataka state. The study area has a literacy rate of 83% (male=86%, female=79%),(4) a high gender related development index (GDI)(5) with a score of 0.714 and a favorable sex ratio of 1022.(4)

The study participants comprised women working in educational institutions, banks, hospitals, and shops as these are the establishments where considerable number of women work. The sample size was calculated based on the expected proportion of harassment faced by working women as 25%,(6) absolute precision of 7% and confidence interval of 95% and 10% non-response error. The final sample size came to 160 women. The list of the banks, schools, hospitals, and shopping centers in the study area were obtained from the municipal corporation office. The study sites were selected by simple random sampling from the list. Finally, the study participants were selected by convenient sampling. The data were collected by means of a pre-tested semi-structured questionnaire after obtaining the written informed consent from the respondents. The questionnaire assessed the respondents’ socio-demographic profile, their perception of harassment, their personal experience and their awareness regarding anti-harassment policies. Socio-economic status was assessed using modified Kuppuswamy’s scale. Data was analyzed using SPSS version 11.5. Chi square test was used for the analysis and *P* value less than 0.05 was considered as statistically significant.

## Results

Table 1 depicts the socio-demographic profile of the respondents and the harassment faced by them at workplace. It was seen that the younger respondents faced more harassment compared to the older respondents. There was a linear association which was found to be statistically significant. It was also found that sales girls (80%) followed by nurses (45.7%) faced more harassment compared to school teachers (13.3%) and bank employees (6.1%), this difference was found to be statistically significant. The majority of the respondents who faced harassment were from lower middle and upper lower socio-economic status which was found to be statistically significant. It was also found that respondents with less experience faced more harassment as compared to those with more years of experience at workplace which was found to be statistically significant.

**Table 1:** Socio-demographic characteristics of study participants classified according to harassment status (*N*=160)

Characteristics	Harassment		Chi square	*P* value
	Yes	No		
	No (%)	No (%)		
Age group in years				
Less than 25 years	17 (37)	29 (63)	4.51	0.034
25 to 35 years	15 (31.2)	33 (68.8)		
36 to 45 years	10 (25.6)	29 (74.4)		
46 to 55 years	4 (16.7)	20 (83.3)		
More than 55 years	0 (0)	3 (100)		
Profession				
Bank employees	3 (6.1)	46 (93.9)	49.5	0.001
School teachers	6 (13.3)	39 (86.7)		
Nurses	21 (45.7)	25 (54.3)		
Sales girls	16 (80)	4 (20)		
Socio-economic status				
Upper	11 (22)	39 (78)	21.65	0.001
Upper middle	16 (20)	64 (80)		
Lower middle	16 (64)	9 (36)		
Upper lower	3 (60)	2 (40)		
Working experience in years				
Less than one year	3 (18.8)	13 (81.2)	16.9	0.005
1 to 5 years	24 (47.1)	27 (52.9)		
6 to 10 years	10 (30.3)	23 (69.7)		
11 to 15 years	2 (14.3)	12 (85.7)		
16 to 20 years	5 (27.8)	13 (72.2)		
More than 20 years	2 (7.1)	26 (92.9)		

Table 2 shows that out of 160 working women interviewed, about 28.8% of them were harassed; majority (47.8%) of the respondents were harassed within one year of joining their employment. The perceived reasons for harassment were – them being more efficient than their male colleagues (45.7%), followed by them being beautiful (23.9%). The type of harassment was mostly verbal (67.4%) followed by physical (23.9%) in nature. Among the respondents who were harassed, 52.2% had complained and the most common mode of complaint was spoken (83.4%).

**Table 2:** Reasons and type of harassment faced by the respondents (*N*=46)

Characteristics	No (%)
Harassed	
Yes	46 (28.8)
No	114 (71.3)
Total	160 (100)
When did the harassment start	
1 month ago	10 (21.7)
1 year ago	12 (26.1)
Over a year ago	7 (15.2)
Since joining	17 (37.0)
Perceived reason for harassment	
Beauty	11 (23.9)
More efficient than others	21 (45.7)
Threatened by skill	7 (15.2)
Less efficient than others	4 (8.7)
Prefer women unemployment	3 (6.5)
What was the harassment	
Spoken	31 (67.4)
Physical	11 (23.9)
Others	4 (8.7)
When did the harassment occur and how often	
Starting few days since joining	8 (17.4)
Continuously	8 (17.4)
Occasionally	30 (65.2)
Complaint of harassment	
Yes	24 (52.2)
No	22 (47.8)
Method of complaint (*N*=24)	
Verbal	20 (83.4%)
Written	02 (8.3%)
Written and possess a copy	02 (8.3%)

## Discussion

In our study, we found that about 28% of the study subjects had experienced some form of harassment, out of which 37% were less than 25 years of age. This could be because the younger girls are more vulnerable and are unaware about the job requirements, or it could be due to the fear of losing their job or a hostile atmosphere in their workplace if they complain. This coincides with a study done in Denmark, where the occupations which were most exposed to the threat of physical violence were nurses, followed by health care workers and teachers.(6)

Among the 48 (28.8%) women harassed, 22 (48.8%) revealed that they had been harassed within a year of joining their jobs. This recent harassment could be explained by the fact that when the women join their new jobs, they are ignorant of their right to complain about harassment and afraid of losing their jobs. The reasons perceived by women as the cause of harassment were them being more efficient in their jobs (45.7%) than their male counterparts, followed by them being beautiful (23.9%). This could be attributed to the fact that males feel less secure about their jobs when their female colleagues are good looking or are more efficient. This also reflects our male dominated society where people still think that males are superior to females be it even in the workplace. Majority (67.3%) of harassment were verbal in manner whereas 22.7% were physical. Similar results were found in a study done in Croatia among school teachers.(7) Complaints were lodged in 52.2% of the cases; mostly to their higher authorities of which only 8.3% were written. Other studies also substantiate these findings.(8–10) It was found that action was taken only in 15.2% of the cases whereas 41.3% of the complaints were ignored. The fewer written complaints explain the hesitancy of the women to complain since action was taken in only a few cases and it could also be because the women felt it would create a bad working atmosphere.

## Conclusion

Our study gives an insight into the depth of the workplace harassment among women, which is on the rise because of the increase in number of working women. Harassment is a serious problem that must be addressed by the government in order to ensure a safe working environment for women.
